# Carvacrol attenuated neuroinflammation, oxidative stress and depression and anxiety like behaviors in lipopolysaccharide-challenged rats

**DOI:** 10.22038/AJP.2022.20005

**Published:** 2022

**Authors:** Hossein Salmani, Zahra Hakimi, Zohre Arab, Narges Marefati, Mohammad Reza Mahdinezhad, Abolfazl RezaeiGolestan, Farimah Beheshti, Mohammad Soukhtanloo, AmirAli Mahnia, Mahmoud Hosseini

**Affiliations:** 1 *Student Research Committee, Jiroft University of Medical* *Sciences, Jiroft, Iran*; 2 *Student Research Committee, Mashhad University of Medical Sciences, Mashhad, Iran*; 3 *Faculty of Medicine, Ghalib University, Herat, Afghanistan*; 4 *Neuroscience Research Center, Mashhad University of Medical Sciences, Mashhad, Iran*; 5 *Department of Physiology and Medical Physics, Faculty of Medicine, Baqiyatallah University of Medical Sciences, Tehran, Iran*; 6 *Applied Biomedical Research Center, Mashhad University of Medical Sciences, Mashhad, Iran*; 7 *Neuroscience Research Center, Torbat Heydariyeh University of Medical Sciences, Torbat Heydariyeh, Iran*; 8 *Department of Physiology, School of Paramedical Sciences, Torbat Heydariyeh University of Medical Sciences, Torbat Heydariyeh, Iran *; 9 *Department of Biochemistry, School of Medicine, Mashhad University of Medical Sciences, Mashhad, Iran*; 10 *Psychiatry and Behavioral Sciences Research Center, Mashhad University of Medical Sciences, Mashhad, Iran*

**Keywords:** Inflammation, Anxiety, Depression, Carvacrol, Oxidative stress

## Abstract

**Objective:**

The beneficial effect of carvacrol on neuroinflammation, oxidative damage of brain tissue, and depressive- and anxiety-like behaviors after lipopolysaccharide (LPS) administration were evaluated in rats.

**Materials and Methods:**

Vehicle (1% Tween 80), 1 mg/kg of LPS, and carvacrol (25, 50, or 100 mg/kg administered prior to LPS) were injected and behavioral and biochemical tests were done.

**Results:**

The results of forced swim test revealed that carvacrol attenuated immobility time and increased activity and climbing times (p<0.05 to p<0.001). The results of elevated plus maze also revealed that treatment by carvacrol prolonged the open arms time and entries and decreased the time and entries in the closed arms (p<0.05 to p<0.01). Carvacrol enhanced crossing, time, and traveled distance in the central segment of the open field and increased total crossing and distance while attenuating the peripheral zone time (p<0.05 to p<0.001). All doses of carvacrol attenuated TNF- α (tumor necrosis factor α) and NO (nitric oxide) in the brain (p<0.01 to p<0.001). The 50 and the 100 mg/kg doses of carvacrol decreased malondialdehyde (p<0.001 for both), and the 100 mg/kg dose of carvacrol increased the content of the thiol (p<0.001).

**Conclusion:**

In conclusion, carvacrol improved the behavioral consequences of LPS challenge and attenuated neuroinflammation and brain tissue oxidative stress in rats.

## Introduction

It is well documented that systemic inflammation is accompanied by a mirror inflammation in the central nervous system (CNS), which later influences brain function. Neuroinflammation, which occurs after systemic inflammation, is reported to be related to the activation of microglia and infiltration of immune cells from peripheral circulation into the brain parenchyma (Miller and Raison, 2015 ▶). Activation of microglia is followed by emergence of high levels of a range of inflammatory mediators such as interleukin (IL)-1β, IL-6, interferon-γ (IFN-γ), tumor necrosis factor α (TNF-α), and other secondary mediators (Patterson, 2015 ▶). Some behavioral changes have been reported to be accompanied by neuroinflammation which are collectively named sickness behavior. It is a part of the host's homeostatic defense to ﬁght against inflammation. Sickness behavior is marked by fever, fatigue, anhedonia, reduced appetite, cognitive impairment, sleep disturbances, somnolence, depression, anxiety-like behaviors, and reduced social and motor activity (Cunningham, 2013 ▶; Holmes, 2013 ▶; Roth et al., 2004 ▶; Teeling and Perry, 2009 ▶). A close relationship between depression and neuroinflammation has also been suggested. Depression is not age-dependent and it could happen in all ages among children, adolescents, adults, and the elderly. It is a common and serious mood disease in which, the patient is repeatedly sad, and has a low mood, and a low level of activity (Zung, 1965 ▶). The concentration of inflammatory cytokines in the peripheral blood of depressed people has been reported to be increased along with signs such as lack of pleasure, reduced activity, and cognitive impairment (Raison et al., 2006 ▶). Recent findings have shown that inflammation and the produced cytokines such as IL-6 and TNF-α affect the HPA (hypothalamus-pituitary-adrenal) axis and the release of corticotrophin-releasing hormone (CRH) and increase blood cortisol. Pro-inflammatory cytokines also decrease the monoamines, especially serotonin in the brain and cause depression (Furtado and Katzman, 2015 ▶; Miller et al., 2009 ▶).

In animal experiments, systemic lipopolysaccharide (LPS) injection has been repeatedly used to induce neuroinflammation. LPS injection in rats and mice has been reported to be followed by sickness behaviors which seems to be primarily mediated through increases of inflammatory mediators such as TNF-α, IL-6, and IL-1β in the brain (Holmes, 2013 ▶; Salmani et al., 2020 ▶). For fever induction, IL-6 is required, whereas IL-1β and TNF-α are known to be involved in behavioral response (Thomson et al., 2014 ▶). Anxiety- and depressive-like behaviors have been frequently reported after systemic LPS injection (Huang et al., 2008 ▶). On the other hand, anti-inflammatory drugs have been well known to exert anti-depression and anxiolytic effects (Beheshti et al., 2020 ▶). In addition, the drugs used to treat brain disorders, including depression, show anti-inflammatory properties (Bakhtiari-Dovvombaygi et al., 2021 ▶; Hosseini et al., 2021 ▶; Khazdair et al., 2015 ▶; Marefati et al., 2021 ▶; Zhao et al., 2020 ▶). Some plants and their components have also been shown to have beneficial effects on cognitive impairments, depression, and anxiety, probably through ameliorating inflammation and oxidative stress (Arab et al., 2020 ▶; Khazdair et al., 2015 ▶; Norouzi et al., 2016 ▶; Samarghandian et al., 2018 ▶). In this matter, the protective effects of oregano, *Zataria multiflora*, and thyme, which contain a considerable amount of carvacrol, against CNS disorders have been suggested (Arab et al., 2020 ▶).

Carvacrol is a compound found in the numerous aromatic plants such as oregano and thyme (Silva et al., 2012 ▶). Carvacrol has antioxidative and anti-inflammatory properties (Amin et al., 2021 ▶; Amin et al., 2020b ▶; Ghorani et al., 2021a ▶). The positive effects of carvacrol on respiratory system disorders have also been reported (Alavinezhad et al., 2017 ▶; Amin et al., 2020a ▶; Amin et al., 2021 ▶; Boskabady et al., 2016 ▶; Ghorani et al., 2021a ▶; Ghorani et al., 2021b ▶; Ghorani et al., 2019 ▶; Khazdair et al., 2018 ▶; Khazdair and Boskabady, 2019a ▶; Khazdair and Boskabady, 2019b ▶). Carvacrol has been reported to reduce inflammatory indicators, including IL-1β, IL-6, TNF-α, and prostaglandin E2 (PGE2), and increase IL-10 production (Amin et al., 2021 ▶; Kianmehr et al., 2019 ▶; Kianmehr et al., 2016 ▶; Kianmehr et al., 2017 ▶; Lima Mda et al., 2013 ▶). The hepatoprotective effects of carvacrol have also been suggested (El-Gendy et al., 2021 ▶; Mortazavi et al., 2021 ▶). According to previous studies, carvacrol has anti-anxiety and anti-depressant properties through the gamma-aminobutyric acid (GABA)ergic pathway (Melo et al., 2010 ▶). By stimulating the serotonergic and noradrenergic systems, carvacrol also has an anti-depressant effect (Melo et al., 2011 ▶). Considering the role of inflammation in nervous system disorders, this study was designed to evaluate the effects of carvacrol on neuroinflammation, oxidative stress, and depressive- and anxiety-like behaviors after lipopolysaccharide (LPS) administration in rats.

## Materials and Methods

**Animals, grouping and drugs**

Fifty rats (weighing 230±20 g) were maintained in a standard environment (temperature 23±1°C with a periodic light/dark cycle). Food and tap water were available for the rats. The Committee on Animal Research of Mashhad University of Medical Sciences fulfilled working with animals in accordance with approved procedures (Approval ID: IR.MUMS.MEDICAL.REC.1398.809). The rats were assigned into the following groups (*n*=7-10), (i) Control, (ii) LPS, (iii) LPS-CAR25 (received carvacrol 25 mg/kg), (iv) LPS-CAR50 (received carvacrol 50 mg/kg), and (v) LPS-CAR100 (received carvacrol 100 mg/kg). The number of animals within each group was selected based on previous works (Arab et al., 2020 ▶).

The experiments were done during seven days. During the first three days, the animals were intraperitoneally (i.p.) treated by 25, 50, and 100 mg/kg of carvacrol (dissolved in 0.1% Tween 80) (Sigma-Aldrich Chemical Co.) in groups iii, iv, and v or its vehicle in the groups i and ii without LPS injection. On days 4-6, rats were injected i.p*.* with carvacrol in groups iii-v or its vehicle in control or LPS groups 30 min prior to LPS or saline injection. These days, the animals were i.p. injected with 1 mg/kg LPS (Sigma-Aldrich Chemical Co.) (in the LPS, LPS-CAR25, LPS-CAR50, and LPS-CAR100 groups) or equal volume of saline (in the Control group) two hours before the behavioral tests. Finally, the rats were sacrificed on the 7^th^ day. 

**Behavioral test**

**Forced swim test**

Forced swim test (FST) was used to evaluate depressive-like behavior in rats. The apparatus was a chamber filled up to 0.3 m with water (22 to 24℃). The animal was placed in the water for 5 min, and immobilization, and active and climbing motions were evaluated (Arab et al., 2020 ▶; Aryanezhad et al., 2021 ▶; Norouzi et al., 2016 ▶; Patro et al., 2016 ▶). 

**Elevated plus maze**

The elevated plus maze (EPM) was used to investigate anxiety-like behavior. This apparatus includes two open and two closed arms facing each other. The test was done as previously reported (Arab et al.,2020 ▶; Norouzi et al., 2016 ▶; Cioanca et al., 2014 ▶).

**Open field**

The open field equipment was a square box (100×100×40 cm), and the floor of the apparatus was divided into two smaller segments called central and peripheral zones. The test was done as previously reported ( Arab et al.,2020 ▶; Norouzi et al., 2016 ▶; Hilakivi-Clarke et al., 1990 ▶).

**Tissue collection**

The day after the behavioral tests, the rats were deeply anesthetized using ketamine and xylazine injection, and the brains were removed and stored in -20℃ for MDA, thiol, TNF-α, and NO metabolite levels assessment. 

**Biochemical measurements**

The concentration of TNF-α was measured using an ELISA kit (Kermania Company, Kerman, Iran). The procedure provided by the company was followed. To estimate lipid peroxidation in the brain, MDA was measured. The method was previously reported (Hakimi et al., 2020 ▶). The brain thiol content was also measured using 5, 5'-dithio-bis-(2-nitrobenzoic acid) (DTNB) as a reagent. The method was previously reported (Hakimi et al., 2020 ▶). The level of the metabolite of NO was estimated using the Griess reagent (Promega Corporation, USA). The company's instructions were considered for measurement. 

**Statistical analyses**

SPSS 16.0 was used to analyze the collected data and data are reported as mean±S.E.M. One-way analysis of variance (ANOVA), followed by Tukey's *post hoc* test, was used to analyze the significant differences among the groups. A p less than 0.05 was considered a significant difference.

## Results

**Carvacrol improved rats' performance in the FST**

**Figure 1:**
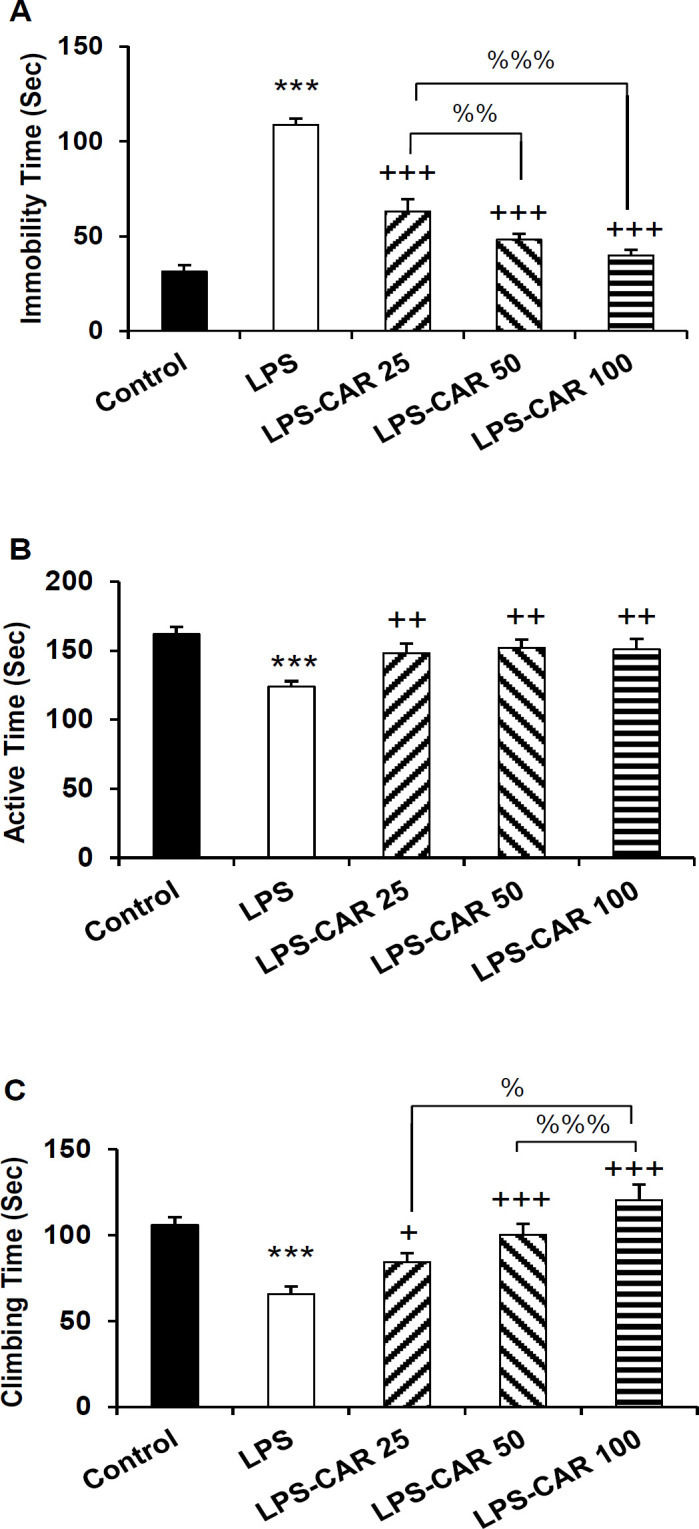
The data of immobility time (A), active time (B), and climbing time (C) in the FST. ***p<0.001 shows the difference between the LPS and control groups. ^+^p<0.05, ^++^p<0.01 and ^+++^p<0.001 show the difference between carvacrol-treated groups and LPS group. ^%^p<0.05, ^%%^p<0.01 and ^%%%^p<0.001 show the difference among the carvacrol-treated groups. LPS: lipopolysaccharide and CAR: carvacrol

The FST results revealed that in the LPS group, immobility time was significantly longer (p<0.001), and active (p<0.001) and climbing (p<0.001) times were significantly shorter compared to the control rats (Figure 1A and B). In the rats pretreated with carvacrol (all doses), the immobility time was significantly decreased (p<0.001 for all doses), and active (p<0. 01) and climbing time (p<0.05 - p<0.001) were increased compared to the LPS group (p<0.001 for all doses). In addition, carvacrol 50 and 100 mg/kg more effectively decreased the immobility time in comparison to the 25 mg/kg dose (p<0.01- p<0.001) (Figure 1A and B). 

**Carvacrol improved** **rats' performance in the EPM test**

**Figure 2:**
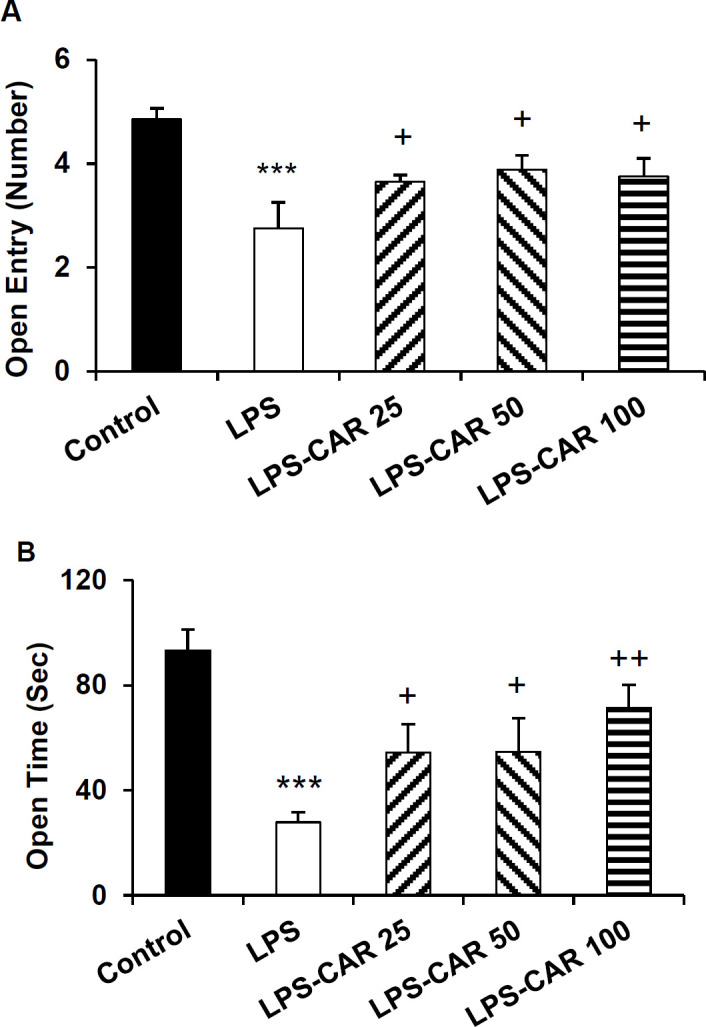
Open arms entries (A) and the time spent in the open arms (B). ***p<0.001 shows differences between the LPS and control groups. ^+^p<0.05 and ^++^p<0.01 show differences between the carvacrol-treated groups and the LPS group. LPS: lipopolysaccharide and CAR: carvacrol

**Figure 3:**
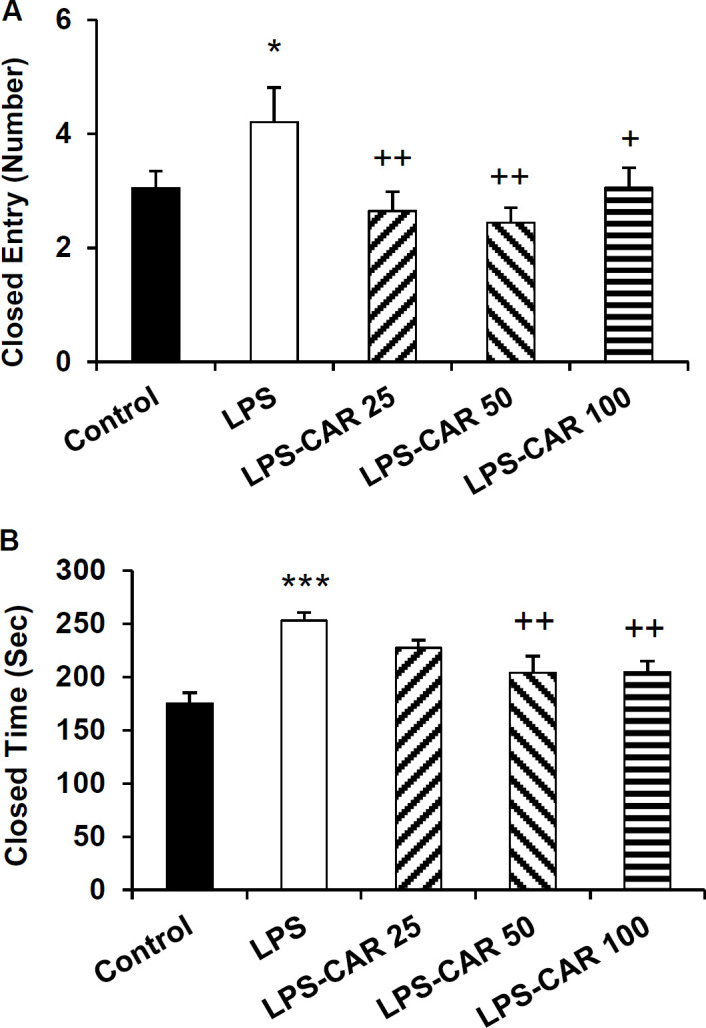
Closed arms entries (A) and the time spent in the closed arms (B) in the EPM. *p<0.05 and ***p<0.001 show differences between the LPS and control groups. ^+^p<0.05 and ^++^p<0.01 show differences between the carvacrol-treated groups and the LPS group. LPS: lipopolysaccharide and CAR: carvacrol

In the EPM test, LPS injection led to a decrease in the open arms entries and time spent (p<0.001 for both) and an increase in the closed arms entries and time (p<0.05 and p<0.001, respectively) (Figures 2 and 3). Carvacrol, at all doses, increased the frequency of entry (p<0.05 for all doses) and time spent in the open arms (p<0.05 for the lowest and the medium doses and p<0.01 for the highest dose) (Figures 2A and B). Compared to the LPS-injected group, carvacrol in all three doses decreased the frequency of closed arm entries (p<0.01 for the 25 and 50 mg/kg doses and p<0.05 for the 100 mg/kg dose) (Figure 3A). In addition, the medium and the highest doses of carvacrol attenuated the time spent in the closed arms (p<0.01 for both) (Figure 3B). Overall, no significant differences were observed among the different doses of carvacrol with respect to rats' performance in the EPM.

**Carvacrol improved rats' performance in the open field**

**Figure 4:**
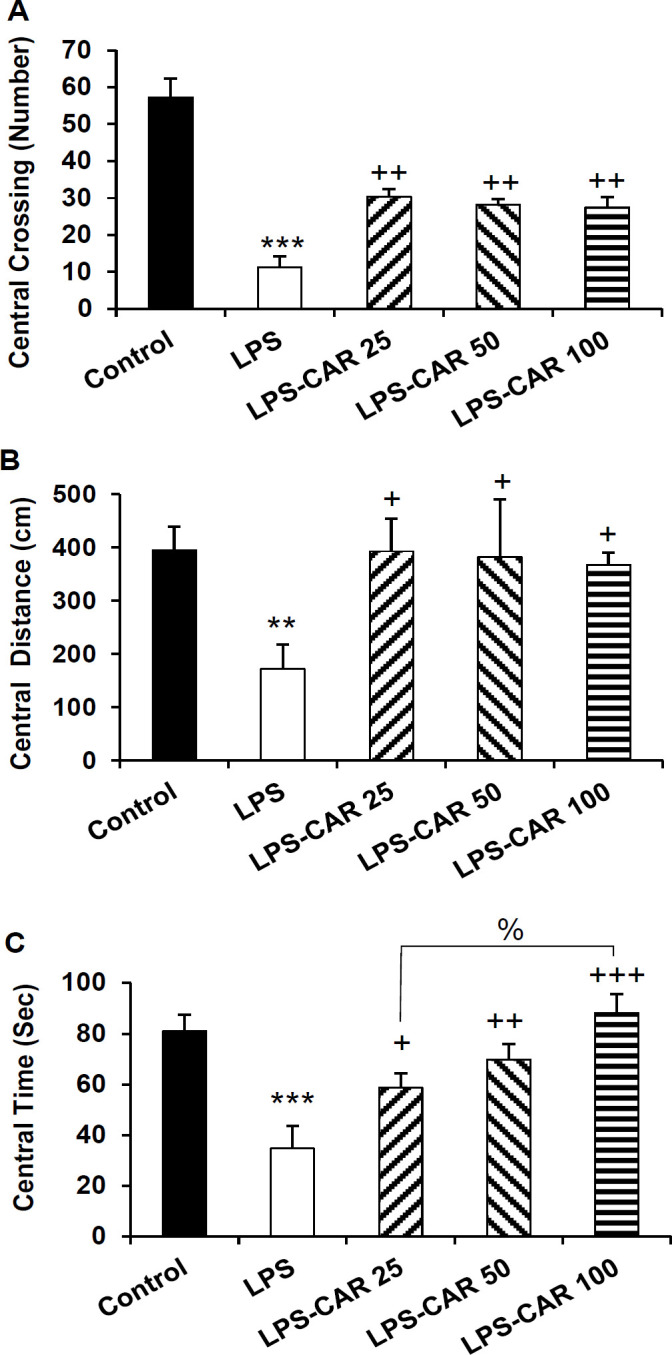
The data of frequency of line crossing (A), traveling path (B), and spending time (C) in the central area of the open field apparatus. **p<0.01 and ***p<0.001 show differences between the LPS and the control groups. ^+^p<0.05, ^++^p<0.01, and ^+++^p<0.001 show differences between the carvacrol-treated groups and the LPS group,^ %^ p<0.05 shows the difference between the 100 mg/kg carvacrol and 25 mg/kg carvacrol. LPS: lipopolysaccharide and CAR: carvacrol

In the open field, the LPS-treated rats crossed less frequently the central area (p<0.001), traveled a shorter distance (p<0.01) and spent a shorter time (p<0.001) than the control rats. In comparison to the LPS group, carvacrol (all three doses) increased the frequency of central area crossing (p<0.01 for all doses), traveled distance in the central area (p<0.05 for all doses), and the time spent in the central zone (p<0.05 for LPS-CAR25, p<0.01 for the LPS-CAR50 and p<0.001 for LPS-CAR100) of the open field (Figures 4A, B and C). 

**Figure 5:**
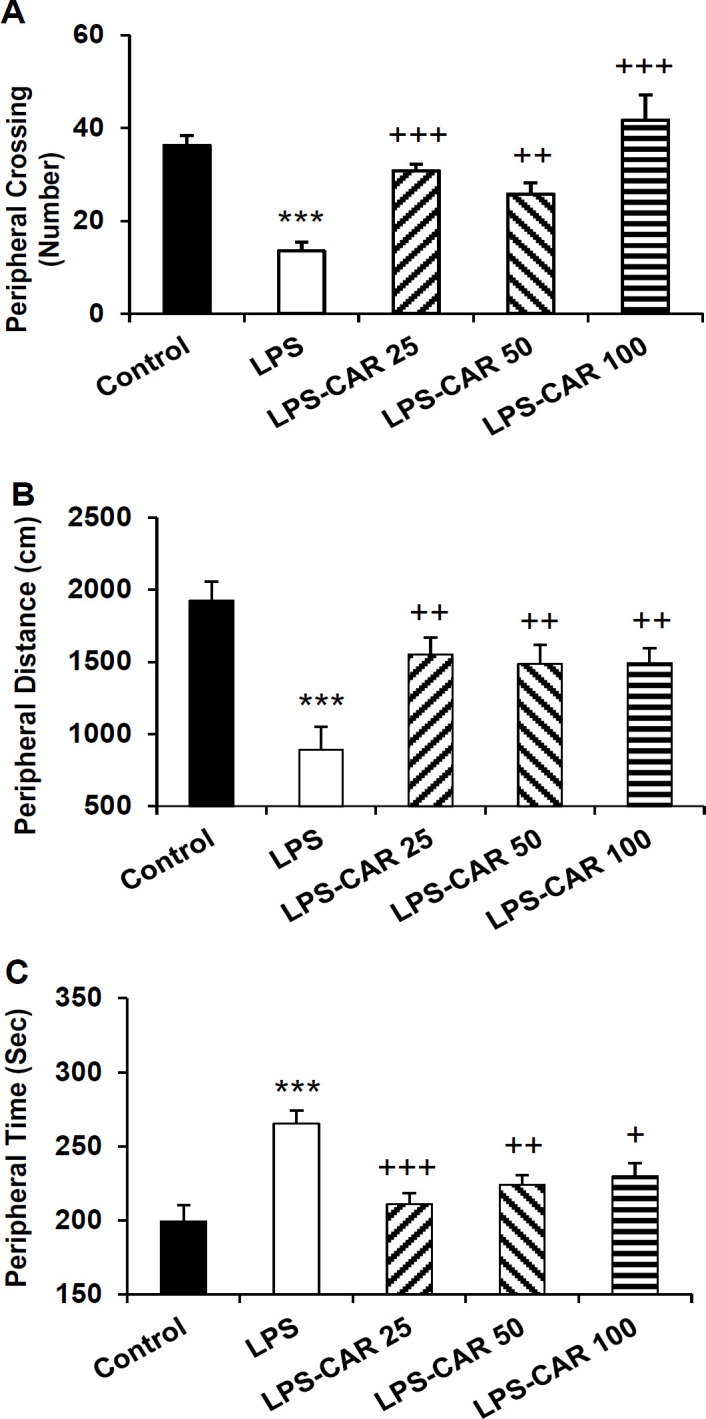
The data of frequency of line crossing (A), traveling path (B), and spending time (C) in the peripheral area of the open field apparatus. ***p<0.001 shows differences between the LPS and control groups. ^+^p<0.05, ^++^p<0.01, and ^+++^p<0.001 show significant differences between the carvacrol-treated groups and the LPS group. LPS: lipopolysaccharide and CAR: carvacrol

As Figure 5 shows, in the LPS-injected group a significant decrease in peripheral area crossing (p<0.001) and traveled distance (p<0.001) and a significant increase in the peripheral zone time (p<0.001) was shown (Figures 5 A, B, and C). Pretreatment with carvacrol 25, 50, and 100 mg/kg doses increased the crossing number (p<0.001, p<0.001, and p<0.01 respectively) and traveled distance (p<0.01 for all doses) in the peripheral zone and reduced the time of the peripheral zone (p<0.001, p<0.01, and p<0.05, respectively). 

**Figure 6:**
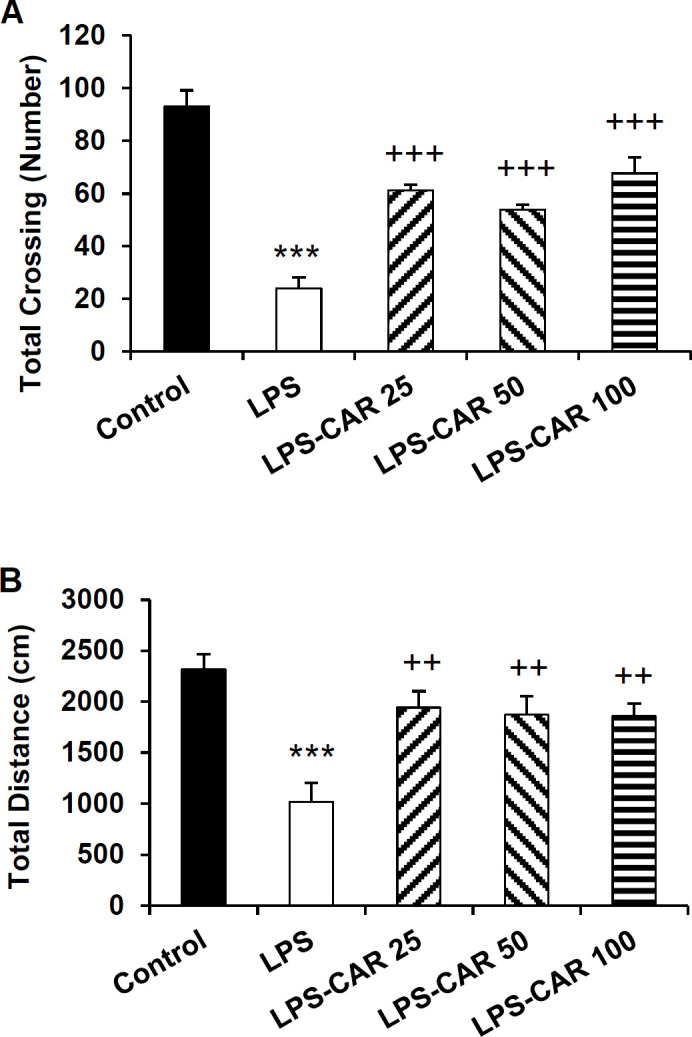
The data of the frequency of line crossing (A) and traveling path (B) in the total area of the open field apparatus. ***p<0.001 shows differences between the LPS and control groups. ^++^p<0.01 and ^+++^p<0.001 show differences between the carvacrol-treated groups and the LPS group. LPS: lipopolysaccharide and CAR: carvacrol

Additionally, in the LPS group, the total traveled distance (p<0.001) and the total crossing number (p<0.001) were lower than the control (Figures 6A and B). Carvacrol in 25, 50, and 100 mg/kg doses increased the total crossing number (p<0.001 for all doses) and the total traveled distance (p<0.01 for all doses) compared to the LPS-injected rats. 

**Carvacrol decreased TNF-α and NO metabolites levels in the brain**

**Figure 7:**
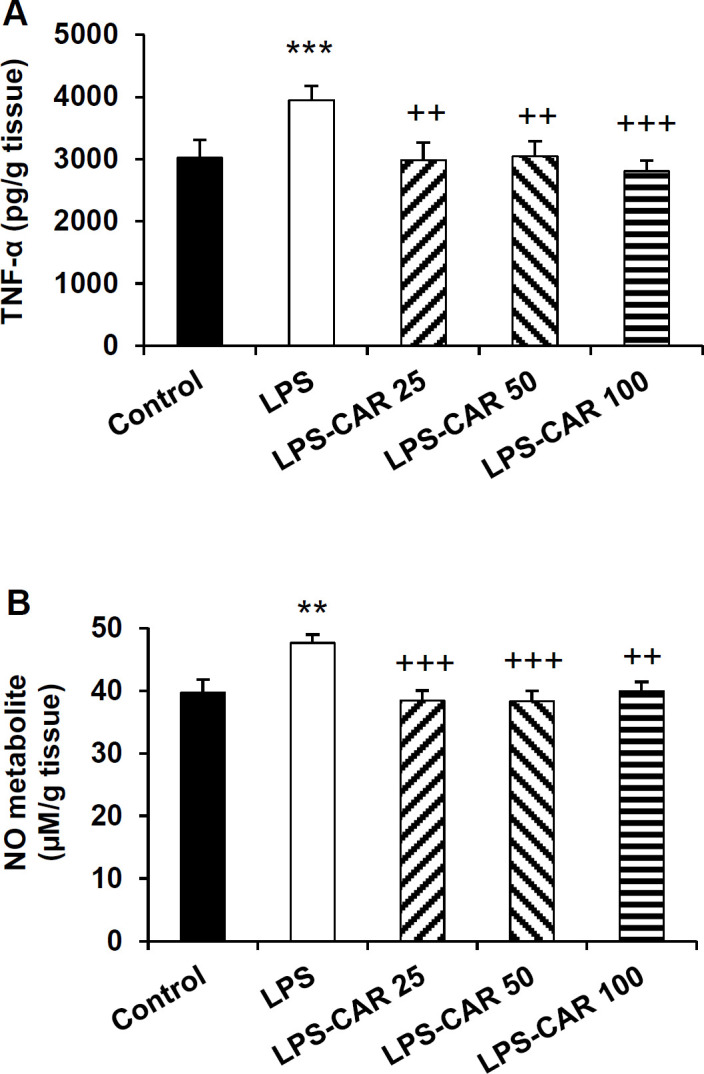
The data of TNF-α (A) and NO metabolite (B) in the brain. **p<0.01 and ***p<0.001 show differences between the LPS group and the control group. ^++^p<0.01 and ^+++^p<0.001 show differences between the carvacrol-treated groups and the LPS group. LPS: lipopolysaccharide, CAR: carvacrol. TNF-α: tumor necrosis factor α and NO: nitric oxide

LPS injection significantly increased the level of TNF-α (p<0.001) and NO metabolites (p<0.01) levels in the brain tissue of the LPS-injected rats compared to the control rats. Carvacrol at all three doses (25, 50, and 100 mg/kg) decreased TNF-α levels (p<0.01, p<0.01, and p<0.001, respectively) (Figure 7A). Additionally, NO concentration in the brain tissue of carvacrol-treated rats (all doses of carvacrol) was lower than the LPS-injected rats (p<0.001, p<0.001, and p<0.01, respectively) (Figure 7B). There was no difference in the NO and TNF-α levels among the different doses of carvacrol (Figure 7). 

**Carvacrol decreased MDA and increased thiol content in the brain**

**Figure 8:**
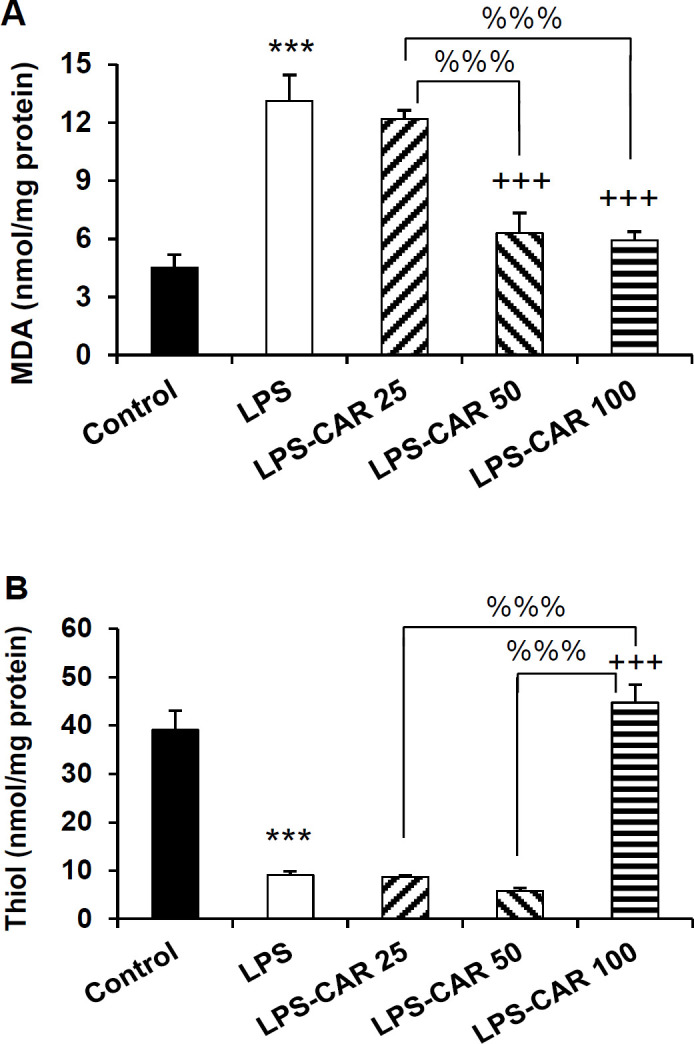
MDA (A) and thiol content (B) in the brain. ***p<0.001 show differences between the LPS group and the control group. ^++^p<0.01 and ^+++^p<0.001 show differences between the carvacrol-treated groups and the LPS group. ^%%%^p<0.001 shows the significant differences among the carvacrol-treated groups. LPS: lipopolysaccharide, CAR: carvacrol and MDA: Malondialdehyde

The biochemical assessments showed that the injection of LPS increased brain tissue MDA concentration (p<0.001) and reduced thiol content (p<0.001) (Figure 8A and B). In comparison with the LPS group, carvacrol (50 and 100 mg/kg but not 25 mg/kg doses) decreased MDA concentration (p<0.001 for both). MDA level was significantly lower in the brain tissue of 50 and 100 mg/kg carvacrol groups compared to the 25 mg/kg carvacrol group (p<0.001 for both) (Figure 8A). In the brain tissue of the 100 mg/kg-treated rats, the thiol content was higher than that in the LPS, LPS-CAR25, and LPS-CAR50 groups (p<0.001 for all) (Figure 8B). 

## Discussion

In the present study, carvacrol treatment reduced anxiety and depressive-like behaviors in the LPS-injected rats and decreased oxidative stress and neuroinflammation in the brain. It is well documented that systemic inflammation is followed by activation of the immune system and induction of depression and anxiety (Dantzer et al., 2008 ▶). In response to systemic LPS administration, microglial cells release pro-inflammatory indicators, such as IL-1β, TNF-α, IL-6, NO, and reactive oxygen species (ROS) in the brain regions such as the hippocampus and hypothalamus (Puffenbarger et al., 2000 ▶). The findings of our current study showed that TNF-α and NO in the brain tissue significantly increased after LPS injection, which indicates a neuroinflammation status. Our results also revealed that neuroinflammation triggered by LPS injection affected the performance of the rats in FST, EPM, and OF tests. 

The FST is a common method to assess depressive-like behavior in rodents. In our current research, the LPS-injected rats had a longer immobility time while shorter active and climbing times. These results confirm a depressive-like behavior as a manifestation of sickness behavior due to inflammation. A close association between inflammation and depressive-like behavior has been frequently suggested (Miller and Raison, 2015 ▶). For instance, treatment of patients by IL-2 and interferon-gamma is followed by the symptoms of depression (O'Connor et al., 2009 ▶). Interestingly, patients suffering from depression have all the characteristics of inflammation (Dantzer et al., 2008 ▶). Anti-depressant effects of anti-inflammatory drugs have been reported in experimental animals and also in humans (Ismail and Mirza, 2015 ▶). The findings of other studies also confirmed that treatment with LPS was followed by depressive-like symptoms (Depino, 2015 ▶). 

Sickness behaviors were also characterized by anxiety-like behaviors in the rodents model of inflammation (Azizi-Malekabadi et al., 2015a ▶; Taksande et al., 2015 ▶). EPM and open field tests are well-known tools used to evaluate the anxiety-like behavior in rodents (Azizi-Malekabadi et al., 2015b ▶; Hritcu and Gorgan, 2014 ▶; Seibenhener and Wooten, 2015 ▶; Swiergiel and Dunn, 2007 ▶). LPS injection has been previously shown to be followed by an anxiety-like behavior in rodents as tested in EPM (Hritcu and Gorgan, 2014 ▶). Consistently, we showed that in the EPM and open field tests, LPS-injected rats exhibited poorer performance than the control rats. 

Considering the evidence showing the important role of inflammation in depression, anxiety, and sickness behavior, anti-inflammatory agents are suggested to have some beneficial effects (Kohler-Forsberg et al., 2019 ▶; Yang et al., 2019 ▶). Previous reports indicated antioxidant and anti-inflammatory properties for carvacrol and it is shown that it can reduce the production of a variety of pro-inflammatory agents such as TNF-α, IL-1β, NO, PGE2, and cyclooxygenase (COX)-2 (Landa et al., 2009 ▶; Yanishlieva et al., 1999 ▶; Yin et al., 2012 ▶). Our results indicated that carvacrol attenuated TNF-α and NO levels in the brain, which might reflect an anti-inflammatory effect. 

Our results in the FST test showed that carvacrol-treated animals had shorter immobility time while longer climbing and active times compared to the LPS group. The effects of carvacrol on inflammation-related depressive-like behavior have not been evaluated before; however, in other animal models, it was previously shown that acute treatment by carvacrol reduces the immobility time in the tail suspension and FST tests (Melo et al., 2011 ▶). It was also previously shown that *Zataria multiflora* and some other plants which contain carvacrol, were able to decrease depressive-like behavior induced by LPS (Amiresmaeili et al., 2018 ▶; Arab et al., 2020 ▶). Monoamines are effective in regulating the activity of various brain regions. For example, in the limbic system regions, including the amygdala, hippocampus, and nucleus accumbens, monoamines regulate emotion, and in basal ganglia, they regulate the psychomotor and rewarding process (Mogenson et al., 1980 ▶). It has been reported that the anti-depressant effect of carvacrol is mediated through the interaction with the dopaminergic system (Melo et al., 2011 ▶). Carvacrol inhibits the reuptake of monoamines (Melo et al., 2011 ▶), which may explain the beneficial effects of carvacrol in reducing depressive-like behavior evident in our study; however, more investigations are needed to be done. 

Improved performance was also observed in the carvacrol-treated rats in the EPM and open field tests, confirming possible anxiolytic effects of carvacrol in inflammatory conditions. Using EPM, marble-burying task and light-dark box, it has been previously reported that carvacrol acetate exerted an anxiolytic effect in mice, and this effect was comparable to the effects of buspirone and diazepam (Pires et al., 2013 ▶). The anxiolytic effects of carvacrol have been suggested to be mediated through the GABA pathways (Melo et al., 2010 ▶), which may also play a part in our findings, but it have to be challenged in the further studies.

Previous studies have reported a range of therapeutic properties for carvacrol, including antioxidant effects (Silva and Fernandes, 2010 ▶). In the brain tissue, carvacrol has been reported to protect against tissue oxidative stress induced by LPS injection (Samarghandian et al., 2016 ▶). Carvacrol was previously shown to reduce MDA levels and the metabolites of NO and increase total thiol content and superoxide dismutase in the hippocampus and cortex (Samarghandian et al., 2016 ▶). In the current research, the medium and highest doses of carvacrol reduced MDA levels in the cortex of LPS-treated rats. Carvacrol also increased thiol content in the brain at 100 mg/kg. Considering these findings and the mentioned evidence, it seems that the protective effects of carvacrol may arise from its antioxidant effects; however, more investigations are needed.

Treatment of LPS-injected rats with carvacrol improved their performance on the FST, as well as open field, and EPM tests, and also reduced brain TNF-α, NO, and MDA levels and increased brain thiol content. Considering these results, it seems that the improving effects of carvacrol on anxiety and depressive-like behaviors are due to its anti-inflammatory and antioxidant effects. 

## Conflicts of interest

The authors have declared that there is no conflict of interest.
